# Online supervised versus workplace corrective exercises for upper crossed syndrome: a protocol for a randomized controlled trial

**DOI:** 10.1186/s13063-021-05875-5

**Published:** 2021-12-11

**Authors:** Zohreh Yaghoubitajani, Mehdi Gheitasi, Mohammad Bayattork, Lars Louis Andersen

**Affiliations:** 1 Department of Health and Sports Rehabilitation, Faculty of Sports Science and Health, Shahid Beheshti University, Tehran, Iran; 2 Sport Sciences and Physical Education, Faculty of Humanities Science, University of Hormozgan, Bandar Abbas, Iran; 3 National Research Centre for the Working Environment, Copenhagen, Denmark; 4 Sport Sciences, Department of Health Science and Technology, Aalborg University, 9220 Aalborg, Denmark

**Keywords:** Online corrective exercises, Workplace, Muscle activity, UCS, Pain, Workability

## Abstract

**Background and objective:**

Musculoskeletal disorders (MSDs) including upper crossed syndrome (UCS) are considered as the leading cause of work-related issues worldwide among office workers. Therefore, the present study aims to evaluate the effect of workplace-based versus online-supervised home-based corrective exercises among office workers with UCS.

**Methods and design:**

To this end, 45 subjects within the age range of 30–45 years are randomly assigned to three groups in the present parallel-group, randomized control trial using a pretest-posttest design. These groups include the subjects who receive online-supervised exercise and workplace exercise containing three sessions of intervention for 8 weeks and the control group receives no intervention while performing routine activities. The primary outcome variables are neck-shoulder pain (NSP) and consequent sick leave due to NSP, followed by alignment, workability, and the surface electromyography of upper, middle, and lower trapezius (UT, MT, and LT), sternocleidomastoid (SCM), and serratus anterior (SA) as the secondary variables.

**Discussion:**

The present study seeks to assess the effect of workplace versus online-supervised corrective exercise interventions among 45 office workers suffering from UCS. It is expected to improve and reduce the related symptoms including postural malalignment and imbalance muscles after 8 weeks of corrective exercises. If effective, the findings may lead to adherence and work performance among the office workers, and individuals subjected to UCS can use the benefits of an online-supervised intervention. In addition, the findings may be useful in different workplaces as the evidence for employers to benefit from the reduction in the related costs and side effects of work-related neck/shoulder disorders including work disability, productivity loss, time expense, social insurance, work absenteeism, and treatment costs. Finally, clinicians and corrective exercise therapists can consider it as a clinical based-evidence intervention for their further actions.

**Trial registration:**

Iranian Registry of Clinical Trials IRCT20200729048249N1. Registered on 5 October 2020 (https://en.irct.ir/user/trial/49992/view)

## Background

Nowadays, sedentary work is considered predominant in various parts of the world due to the rapid development of technology and the new nature of work. The workers are exposed to prolonged static posture and repetitive upper limb movements [1, 2] and spend long periods in front of a computer or at a desk in a dorsiflexed position with rounded shoulders [3]. According to Page [4, 5], these prolonged postures may under-activate some muscles while over-activating other muscles leading to joint dysfunction known as “upper crossed syndrome” (UCS). In addition, specific postural changes due to the UCS may decrease glenohumeral stability causing elevated shoulders and scapulae winging. Accordingly, the levator scapula and upper trapezius require increasing activation in order to maintain glenohumeral centration to compensate for the loss of this stability [4–7]. All these alterations may be associated with work-related neck/shoulder disorders (WNSDs) although the casualty of the association between computer use and pain is unknown [8].

As one of the main concerns of public health, WNSDs may be related to pain and impaired physical functions causing musculoskeletal complaints and affecting work performance among the office workers [9]. Further, WNSDs with annual prevalence rates of 27–48% influence the musculoskeletal system leading to numerous work-related disorders [10, 11]. Regardless of the actual cause of pain, musculoskeletal pain in the neck and shoulders increase the risk of long-term sickness absence in white-collar workers [12]. The prevalence of this type of pain was reported relatively high in the neck and shoulders (45.8 and 40.1%, respectively) in Iran [13]. Thus, different employees are at an increased risk of sickness absence. Regarding the major role of WNSDs in both employees and employers, as the most common reasons for work disability, sick leaves, and early retirement, it is considered as one of the most significant current discussions due to the cost accompanying treatment, production loss, and work absenteeism [14–16]. Thus, it has become one of the most serious challenges in occupational health for reducing the financial impacts of health-related productivity and labor costs among office workers as a worthwhile business consideration [17].

Neck or shoulder pain symptoms appear to intensify since office workers with UCS generally sit with curved postures, take prolonged constant muscle activity, and perform repetitive job tasks [18, 19]. Pain is considered as the strongest stimulus to central motor programming, which can alter electromyography (EMG) patterns in functional tasks since it has an inhibitory effect on muscle activation [20]. Some clinical studies confirmed that the tenderness of muscles is considered the most common type of neck or shoulder pain in office workers [19, 21, 22].

Due to the relationship between neck or shoulder pain and muscle tenderness, some studies reported that computer workers with neck and shoulder pain might have trapezius myalgia, tension neck syndrome, and cervicalgia [22, 23]. Thus, the majority of the office workers with frequent pain in shoulder and neck experienced tenderness of the upper trapezius muscle [23]. Further, Pietropaoli et al. reported higher EMG activity by more muscle tenderness scores when a general correlation was observed between muscle tenderness and EMG values [24].

Thus, individuals with insidious-onset cervical pain demonstrate poor postural stability more and significantly increased EMG levels in the SCM activity, respectively [25, 26].

Furthermore, due to the affected regions of painful UCS and alteration in muscle activation around the scapula and neck, EMG can be used as a reliable tool to validate the assessment of LT, MT, and SA activity decrease as well as UT activity increase [27–29].

Among office workers, postural changes and movement patterns in the scapula refer to the UCS including postural malalignments and altered muscle activity associated with workability and sickness absence [30]. Such conditions may play an important role in the development of neck and shoulder pain, which can be measured with electromyography as far as physiological measurement is concerned [31]. Meanwhile, balancing and restoring muscle activity by maintaining the alignment (upright body position) can reduce chronic neck pain and induce a more relaxed muscle activity pattern during work [32]. Due to the study population and WRMSDs impacts containing workability and sickness absence, it is evident the importance of managing UCS symptoms along with monitoring muscle activity to decrease the incidence of subsequent impairments [33].

Then, knowledge is highly required regarding effective interventions for relieving WMSD symptoms and preventing the related consequences such as pain, work disability, and sickness absence [34]. In this regard, some studies reported statistically significant positive effects for improving office workers’ workability by increasing physical activities [35]. Additionally, the results of some studies demonstrated that exercise has positive effects on health-related productivity loss and sickness absenteeism among office workers with neck pain in the longer term [36]. In addition, Seeberg et al. found a relationship between forward head posture (FHP) improvement and musculoskeletal pain after therapeutic exercises [34]. Further, the results of some studies indicated that exercises specific to the involved muscles restored malalignment and increased the ability to keep an upright cervical posture during work, as well as improved pain, disability, and the quality of life among office workers [37, 38]. On the other hand, considering that different mechanisms may contribute to abnormal scapular movements, pain, abnormal thoracic posture, and imbalance muscle strength or activation [39], an exercise program can improve neck pain displaying positive and significant alterations in the forward head and protracted shoulder posture, disability, and the timing of superficial neck muscle activation [40, 41]. Finally, some positive effects of exercise interventions are presented for improving postural malalignments based on the majority of studies. However, both the neural and muscular components should be considered to accomplish the best performance [42–45].

Although previous studies have revealed the hopeful results of exercise interventions among different occupational groups, the question that remains is whether such interventions should be implemented under supervision or in the workplace [34, 46]. The performance of worksite exercises in a group may be more motivating for some employees regarding increasing adherence although various barriers exist in this respect during working hours, which may be costly for employers regarding spending time and facilities [46]. Furthermore, although previous literature has demonstrated the positive efficacy of both supervised and unsupervised exercise programs, contradictory results are present about whether supervised or unsupervised exercise is more effective [47–49]. Several studies have simultaneously addressed all the involving factors related to UCS by applying exercise therapy. However, scarce research has focused on some separate areas of the upper body including the neck or shoulder among office workers [19, 40, 43, 50–53]. According to Hall et al. [54], the world is currently experiencing an extraordinary, life-altering challenge due to social distancing and home quarantine recommended by the World Health Organization to minimize the speed of the coronavirus disease (COVID-19). Outdoor physical activities have been postponed in different cases [55, 56] since it is difficult to precisely predict when the COVID-19 pandemic diminishes and communities can return to normal function [57]. Accordingly, online-guided physical activities at home may be a way forward. Thus, studying the effect of workplace versus online-supervised exercises among office workers suffering from WMSDs including UCS is relevant.

### Objectives

Given the explanations mentioned earlier, the present study seeks to evaluate the effect of workplace-based versus online-supervised home-based corrective exercises containing NSP and sick leave due to NSP as the primary objectives.

The secondary objectives include workability, alignment (i.e., neck, shoulder, and thoracic spine angles), and assessing the surface EMG of designated muscles including UT, MT, LT, SCM, and SA among office workers with UCS.

## Method

### Study design

**Fig. 1:**
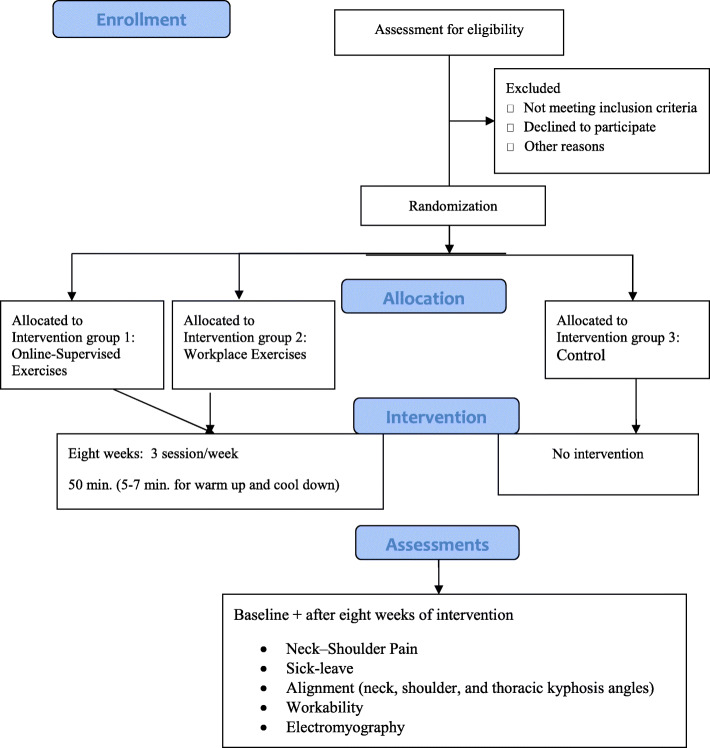
Study flowchart

A parallel-group randomized control trial with a pretest-posttest design is used for the present study. Additionally, the selected subjects are randomly assigned to three groups including two interventions (i.e., online-supervised exercise and workplace exercise receiving 8 weeks of intervention) and a control group with no intervention performing routine activities. In addition, baseline assessments are organized at the Sports Science and Health Laboratory at Shahid Beheshti University, Tehran, Iran, and are repeated after 8 weeks of intervention. The procedure and flow diagram are shown in Table 1 and Fig. 1, respectively. The procedure is followed according to the SPIRIT^1^ guidelines to ensure the apparent and standardized reporting of the trial.

**Table 1:**
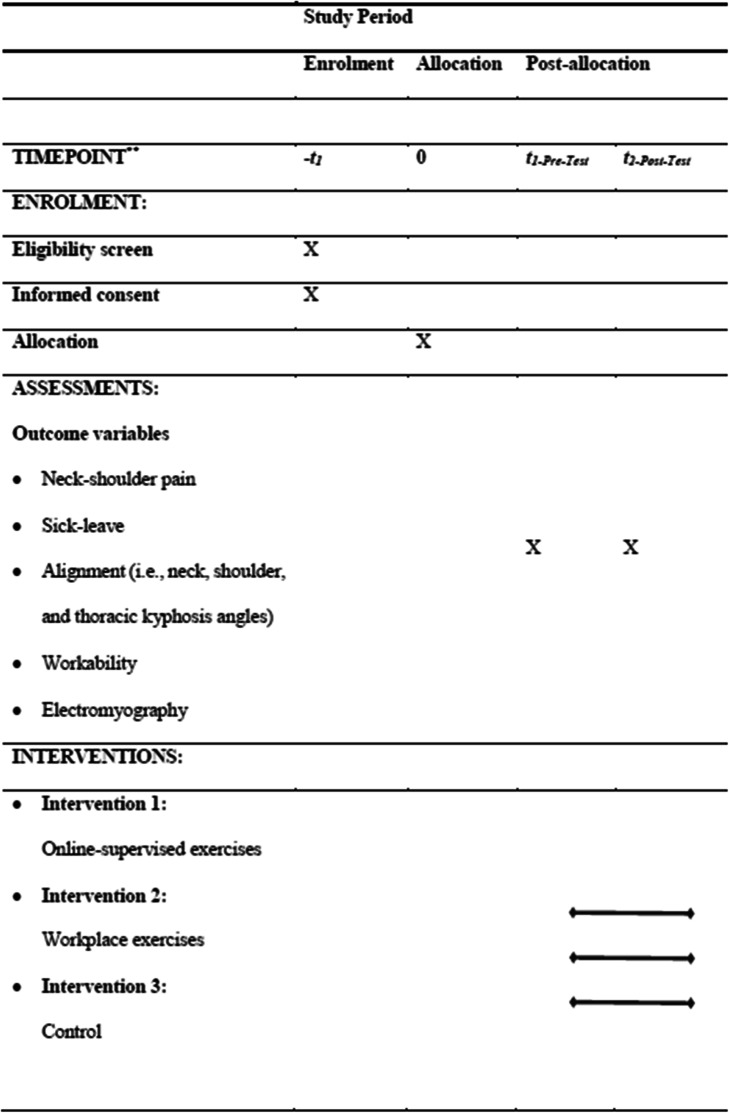
Procedure of the study

### Ethical considerations

Before commencing the study, the procedure including assessments is explained to the subjects, and they are requested to complete and sign an informed consent form. Ethical clearance No. IR.SBU.REC.1399.036 dated 20 June 2020 was issued by the Ethics Committee on the Research at Shahid Beheshti University, Tehran, Iran. Further, IRCT No. IRCT20200729048249N1 dated 5 October 2020 was approved by the Iranian Registry of Clinical Trials (https://en.irct.ir/user/trial/49992/view). Furthermore, deviations from the present protocol are clearly described in the main article with the results of the trial.

### Subjects and eligibility criteria

The corrective exercise specialist (Ph.D.) primarily screens the subjects recruited through invitation letters from private and public organizations in Tehran, Iran, for three main UCS features. Since the presence of scapular dyskinesia may indicate the lack of neuromuscular control, including muscle activation and timing thus, the related tests are performed to evaluate the position and rhythm of the scapula, which plays a significant role in facilitating the upper extremity function among office workers [58–60]. Further, due to any postural alteration influencing the muscle activity, muscle length and muscle strength tests are implemented for UT and pectoral muscles and MT, LT, and deep cervical flexor, respectively [61].

Inclusion criteria for the trial must comply with office workers between 30 and 45 years using a computer or lab-tops most commonly during the working day (about 30 h per week) with at least 5 years of experience [17, 62]. Having alignment alteration includes forward head (≥ 45°), round shoulder (≥ 52°), and round back (≥ 42°) according to previous studies [63, 64]. Marking pain intensity score visual analog scale (VAS) ≥3 in neck and shoulder [17, 58, 65].

Concerning exclusion criteria, those are ineligible if pregnant during the study process, having surgery on the upper extremities during the past year, unable to perform exercise due to any medical conditions, and being in weight out of the normal range (18 ≥ BMI ≥ 25).

Drop out criteria are considered if the subjects attend no post-tests, lose three sequential intervention sessions, and any factors that may affect the study results.

However, the subjects are allowed to discontinue the study at any stage [66]. At the first encounter at baseline, the researchers are asked for permission to contact them in the case of study discontinuation. Accordingly, performing an accurate intention-to-treat analysis of the primary result is possible.

### Randomization

The subjects will be randomized to one of the two treatments and control groups including workplace exercise, online-supervised exercise, and control using https://www.sealedenvelope.com. Randomization will be performed as block randomization with a 1:1 allocation. A sufficient number of the subjects will be recruited according to the sample size calculation to minimize random error. Meanwhile, to ensure the allocation concealment, the randomization code will not be released until the subject has been recruited into the trial, which occurs after all baseline measurements. Therefore, randomization will be performed through a computer-generated sequence for allocation concealment, including concealed, sequentially numbered, sealed, and opaque envelopes. A card inside indicates the allocated group to each subject [67].

The trial investigator will implement the various stages of the randomization process and enrollment and assign the subjects to interventions. The SBU professor assistant will supervise all the procedures implemented by the investigator, including the sequence generation process and allocation concealment mechanism during the study process to ensure that the assignment schedule is unpredictable and locked away from even the person who generated it.

The trial process is not independent of the investigators. The professor assistant (correspondent author) from SBU frequently supervises the procedures for auditing trial conducting a periodic independent review of core trial processes and documents. Auditing contains the participant enrolment, consent, eligibility, and allocation to study groups, adherence to trial interventions and policies to protect participants, including reporting of harms and completeness, accuracy, and timeliness of data collection.

A formal amendment to the protocol will be agreed upon by the Iranian Registry of Clinical Trials (IRCT), including any modifications to the protocol which may impact the conduct of the study, a potential benefit of the subjects, or may affect their safety, including changes of study objectives, study design, study population, sample sizes, study procedures, or significant administrative aspects. Meanwhile, the Ethics Committee on the Research will approve such an amendment at SBU, Tehran, Iran. The trial investigators are responsible for the decision to amend the protocol.

### Intervention

**Fig. 2:**
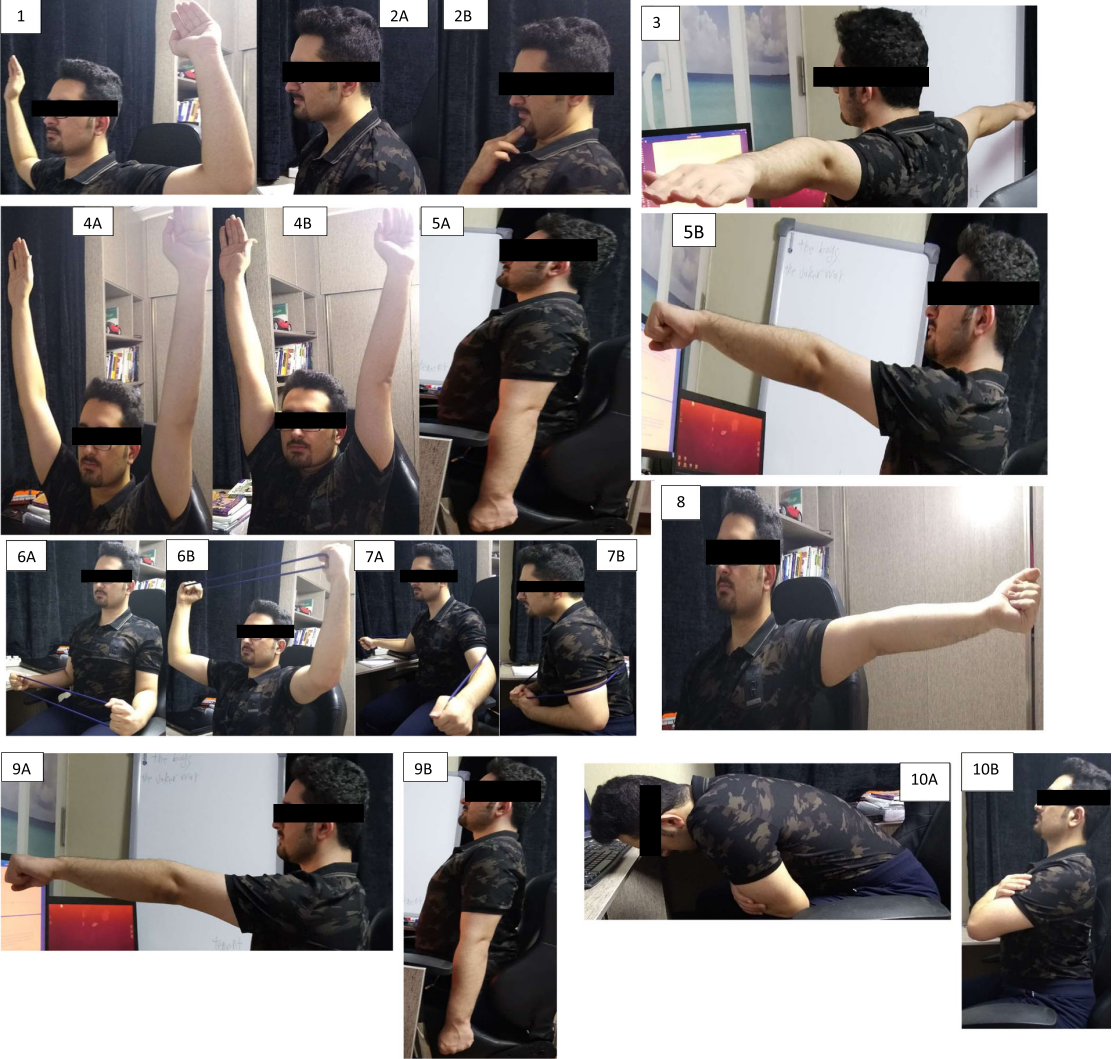
Workplace corrective exercises. **1** Sitting position: arms in a W shape, horizontal abduction with external rotation. **2A**, **2B** Sitting position: chin tuck. **3** Sitting position: scapula retraction and depression with arms in a T shape. **4A**, **4B** Sitting position: scapula retraction overhead and arms overhead. **5A**, **5B** Sitting position: forward flexion starting with the arms parallel with the body. **6A**, **6B** Sitting position: elevation 90° flexed elbows in the scapular plane with 30° external rotation by an elastic band. **7A**, **7B** Sitting position: dynamic hug exercise by the elastic material as resistance performing bilateral, maximum scapular protraction. **8** Sitting position: horizontal arm 90 abduction with external rotation. **9A**, **9B** Sitting position: extension starting with the arm at 90° of forward flexion. **10A**, **10B** Sitting position: thoracic extension exercises

**Fig. 3:**
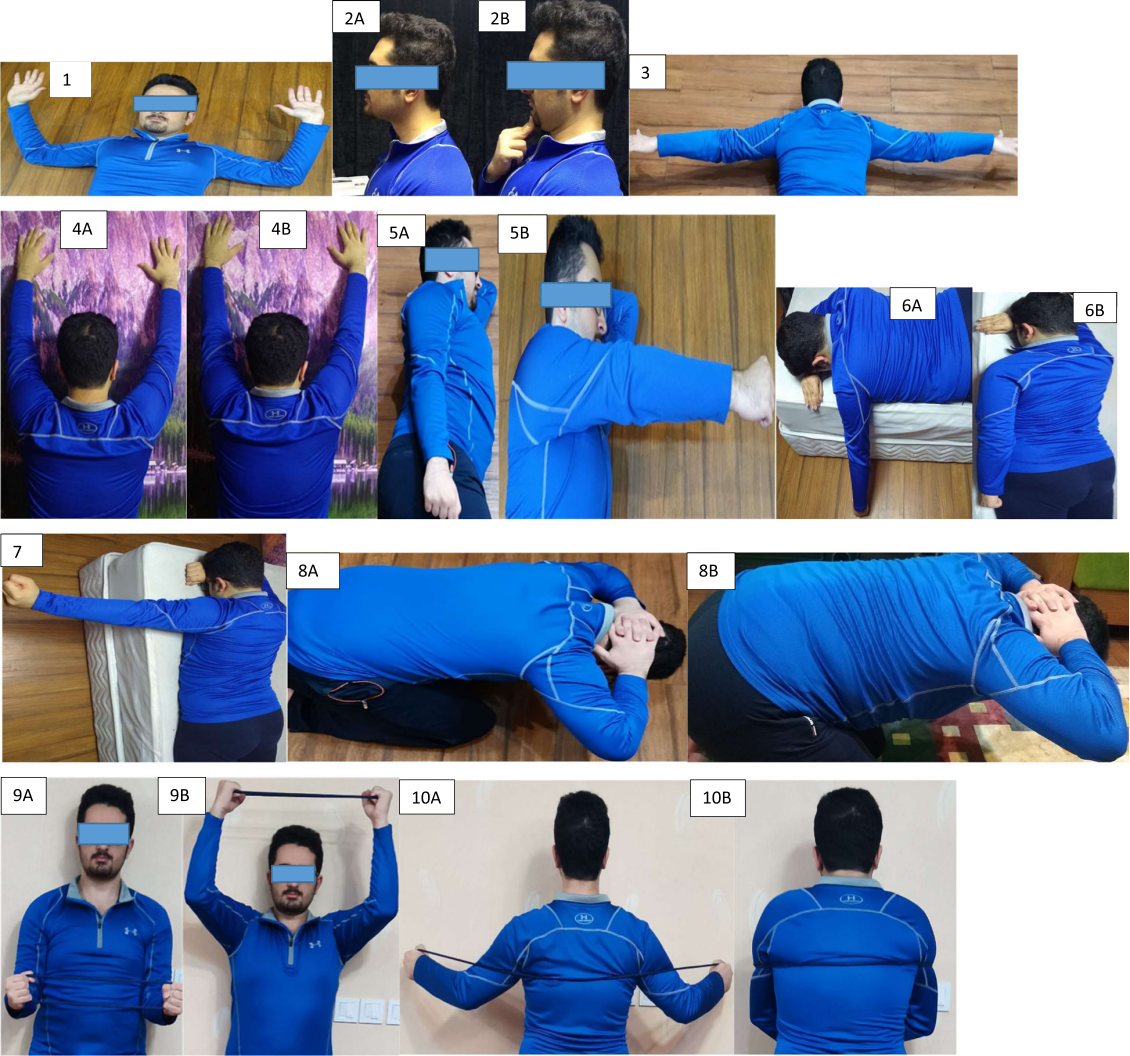
Online-supervised corrective exercises. **1** Supine position: arms in a W shape and horizontal abduction with external rotation. **2A**, **2B** Sitting position: chin tuck. **3** Prone position: scapula retraction and depression with arms in a T shape. **4A**, **4B** Standing position: scapula retraction overhead and arms overhead. **5A**, **5B** Side-lying position: forward flexion starting with the arms parallel with the body. **6A**, **6B** Prone position: extension starting with the arm at 90° of forward flexion. **7** Prone position: horizontal arm 90 abduction with external rotation. **8A**, **8B** Sitting position: thoracic extension exercise. **9A**, **9B** Standing position: Elevation 90° flexed elbows in the scapular plane with 30° external rotation by the elastic band. **10A**, **10B** Standing position: dynamic hug exercise by the elastic material as resistance performing bilateral, maximum scapular protraction

As illustrated in Figs. 2 and 3, an 8-week corrective exercises protocol is taken by two intervention groups considering the UCS features such as alignment, muscle activity, and movement pattern simultaneously. The exercise program is performed three days per week to achieve the best results. Each session lasts nearly 50 min, initiating by 5–7 min of warm-ups and finishing by cool-down, respectively [59]. In addition, the exercises are initiated by three repetitions holding for 10 s using the Borg scale [68] and progress to six repetitions, holding for 25 s based on overload principles and individual characteristics [59, 61]. If reporting pain during the exercise performance, the subjects can discontinue and rest until pain relief, while moderating the exercises accordingly. A qualified corrective exercise instructor supervises the exercise programs of both intervention groups. The online-supervised group includes up to four subjects. Each session is remotely performed and supervised three times a week for 8 weeks in their home environment using real-time desktop videoconferencing software (https://meet.jit.si/) via a laptop computer. Accordingly, subjects can have contact and talk with both the instructor and the other subjects [62]. The workplace group performs all sessions in the worksite without daily face-to-face supervision, although supervision is conducted using diary and telephone interviews. Further, each participant in this group is provided with a detailed written exercise and pictorial descriptions to enhance exercise performance. However, the corrective exercise expert is present once a week during the exercise sessions to provide input and evaluate the progress and ensure that all subjects are exercising safely and correctly [49]. It is noteworthy that the corrective exercises protocol is expected to prevent the undesired lack of scapula stabilization on the thorax diminish neck and shoulder pain. Individuals with shoulder pain have excessive upper trapezius activation and decreased and/or delayed activation of the LT, MT, and SA [66]. Thus, exercises specifically targeting the trapezius and SA muscles are commonly incorporated into rehabilitation programs to optimize the scapular position and motion [69]. Recent studies indicated that the specific training of the neck muscles, such as strengthening deep cervical flexor muscles, could reduce neck pain and improve SCM endurance, which is effective in correcting head and shoulder postures [68, 70]. Therefore, these exercises are recommended to improve the function of muscles in the neck, shoulder, and thoracic for several painful conditions due to their reduced or altered activation. At the follow-up, subjects are asked whether they have experienced any injuries or other adverse events during the training sessions.

The detailed exercise program is available for administration by two interventions groups in Figs. 2 and 3.

### Outcome measures

Before randomization and at baseline, all assessments are performed and repeated after the intervention. NSP and sick leave due to pain are considered as the primary outcome variables, followed by the alignment, workability, and electromyography (EMG) activities of the selected muscles as the secondary variables. In the first part of the questionnaire, the subjects are asked to insert their demographic and social status including gender, age, weight, and height (body mass index = kg/m^2^), work hour/week, years of experience, education level, and marital status [71].

#### NSP intensity

To determine NSP intensity, the subjects are asked through a questionnaire to mark a vertical line on the VAS line at the point which represents their pain intensity in each area (i.e., head, neck, shoulder, and upper back) on a scale ranging from 0 to 10 representing no pain and severe, respectively [71, 72].

#### Workability and sick leave due to pain

Workability is self-assessed using the related questionnaires by a single validated item from the workability index [73, 74]. The subjects respond to one question as to how they rate their current workability according to their capabilities to meet the mental and physical demands of their job, which can still perform in two years. The response scores range from 0 to 10, indicating inability to work and workability with a cut-off point score of ≤7 implying poor workability, respectively [75]. In addition, sick leave due to pain is evaluated using a single item from the validated Outcome Evaluation Questionnaire to obtain data on the number of absence days from work due to pain in muscles or joints within the past month, and response categories ranged from 0 to 31 days [76]. Based on a recent meta-analysis, self-reported sick leave demonstrates good reliability and validity against the records [77].

#### Alignment

To determine the angles for forward head and round shoulder postures, visible landmarks are placed on the ear tragus, the acromion process of the scapula, and the neck seventh vertebra process, as well as the 12th dorsal vertebra of the spinous for measuring the kyphosis angle, respectively [78]. Further, photogrammetry is utilized, asking the subjects to stand laterally and comfortably with bare feet on the flat floor while looking forward. A digital camera is fixed at a distance of 265 centimeters from the subjects, and then three photos are taken from the lateral view [79]. Furthermore, the angles are identified using AutoCAD software (version 2020) connecting a vertical line from the tragus to C7 for determining forward head and continuing to the acromion process for displaying the rounded shoulder angles. To identify the thoracic kyphosis angle, C7 and T12 markers are considered as the starting and ending points of the arch. Previous research indicted the photogrammetry intrarater and interrater reliability ICC 0.98–0.99 and ICC 0.91–0.99 respectively [78, 80–82]. Finally, the mean of three measurements is considered as the alignment angle.

#### EMG

The onset timing and amplitude for the dominant side of the selected muscles (i.e., UT, MT, LT, SCM, and SA) are recorded using EMG, and Matlab software is applied for data analysis [83]. After preparing the skin, electrodes are placed according to the European protocol of SENIAM^2^, and then the reference electrode for each muscle is attached to the nearest bony site of the muscle. Additionally, a maximum voluntary isolated contraction (MVIC) is used to normalize and standardize the data by the root of the mean square (RMS). In addition, the subjects are requested to elevate their hand 30° in the scapular plate without any resistance in three phases (i.e., isometric, concentric, and centric) five times with a 3-s break within each repetition. Then, the mean RMS is calculated based on three of five repetitions, followed by divining the mean RMS by the MVIC value multiplied by 100 to obtain the percentage of muscle activity [84]. The reliability of this method was observed within-day (0.85–0.99) and between-day (0.68–0.93) intraclass correlation coefficient for normalized RMS activity and within-day (0.94 ) and between-day (0.80) intraclass correlation coefficient for time broadness respectively [85, 86].

### Sample size

Based on the results of the previous studies and a pilot study, 11 subjects are calculated for each group using G*Power software (version 3.0.10, Germany) with an alpha level of 0.05, power (1 − β) of 80%, and effect size of 0.66. It should be noted that the effect size was reported in the previous study that investigated the effects of corrective exercises on neck pain between the intervention and control groups [41]. However, to avoid the probability of losing the subjects during the research process, the number is considered 15 in every three groups (*N* = 45 subjects).

### Statistical method and analysis

Data are analyzed using IBM SPSS statistics software, version 24 for Windows, and descriptive statistics are applied to describe the variables considering sig. ≤0.05. Further, data normality is reported based on the Shapiro-Wilk test.

A 3 (group) × 2 (time) mixed-model repeated-measures ANOVA is used to compare all values from the pre-test to each point of the time within each group. Furthermore, the mixed-model repeated-measures ANOVA is applied to analyze within-group changes. Additionally, Bonferroni’s post hoc test for indicating the significance is utilized for any significant difference, and one-way ANCOVA is employed to compare the groups in the post-test with each pre-test value as a covariate. In addition, the effect size is calculated for the magnitude of the difference using the partial *η*2 method as small (0.01 ≤ *η*2 < 0.06), medium (0.06 ≤ *η*2 < 0.14), or large (*η*2 ≥ 0.14). It should be noted that interim analyses were not planned in the present study.

## Discussion

The present randomized control trial is conducted to assess the effect of workplace versus online-supervised corrective exercise interventions among 45 office workers suffering from UCS. The workplace exercise group receives an intervention without the direct supervision of an expert while another group performs the exercise under direct online supervision. These interventions are expected to improve and reduce UCS symptoms containing postural malalignment and imbalance muscles after eight weeks of corrective exercises. Further, it is estimated that the corrective exercises protocol leads to pain relief and an increase in workability in the worksites. Furthermore, the findings may be applied in various workplaces as evidence for those large populations of office workers involving WMSDs where employers can benefit from the actions by decreasing the related costs and side effects (e.g., work disability, productivity loss, time expense, social insurance, work absenteeism, and treatment costs, respectively). In this regard, most studies have only evaluated MSDs in different worksites including pain and work disability concentrating on a separate area (the neck or shoulder) or some specific muscles exclusively. On the other hand, a limited number of studies have considered the associations between malalignment, muscle imbalances, pain, and work disability among office works as a set of disorders named “UCS”. Thus, the results of the present study may lead to the adherence and work performance of office workers who are subject to WMSDs and other individuals with UCS symptoms. Finally, the findings are predicted to elaborate on the effect of workplace exercises with indirect supervision versus direct online-supervision exercises after eight weeks of intervention.

## Trial status

The present trial was registered under No. IRCT20200729048249N1 dated 5 October 2020 and the protocol version No. 49992. At present, the study is in the stage of subject enrollment, and recruitment is expected to begin on 2020.12.20 and complete by 2021.06.20.

## Data Availability

The researchers interested in using the final dataset for scientific purposes may contact the corresponding author.

## Abbreviations

MSDs: Musculoskeletal disorders
UCS: Upper crossed syndrome
NSP: Neck-shoulder pain
UT, MT, and LT: Upper, middle, and lower trapezius
SCM: Sternocleidomastoid
SA: Serratus anterior
WNSDs: Work-related neck-shoulder disorders
EMG: Electromyography
FHP: Forward head posture
VAS: Visual Analogue Scale
MVIC: Maximum voluntary isolated contraction
RMS: Root mean square

## Footnotes

1 Standard Protocol Items: Recommendations for Interventional Trials

2 Surface EMG for non-invasive assessment of muscles
